# Land Cover Classification Model Using Multispectral Satellite Images Based on a Deep Learning Synergistic Semantic Segmentation Network

**DOI:** 10.3390/s25071988

**Published:** 2025-03-22

**Authors:** Abdorreza Alavi Gharahbagh, Vahid Hajihashemi, José J. M. Machado, João Manuel R. S. Tavares

**Affiliations:** 1 Faculdade de Engenharia, Universidade do Porto, Rua Dr. Roberto Frias, 4200-465 Porto, Portugal; up202003516@edu.fe.up.pt (A.A.G.); hajihashemi.vahid@ieee.org (V.H.); 2 Instituto de Ciência e Inovação em Engenharia Mecânica e Engenharia Industrial, Departamento de Engenharia Mecânica, Faculdade de Engenharia, Universidade do Porto, 4200-465 Porto, Portugal; jjmm@fe.up.pt

**Keywords:** Deeplab v3+, ResNet-50, K-medoids clustering, satellite images, multispectral processing

## Abstract

Land cover classification (LCC) using satellite images is one of the rapidly expanding fields in mapping, highlighting the need for updating existing computational classification methods. Advances in technology and the increasing variety of applications have introduced challenges, such as more complex classes and a demand for greater detail. In recent years, deep learning and Convolutional Neural Networks (CNNs) have significantly enhanced the segmentation of satellite images. Since the training of CNNs requires sophisticated and expensive hardware and significant time, using pre-trained networks has become widespread in the segmentation of satellite image. This study proposes a hybrid synergistic semantic segmentation method based on the Deeplab v3+ network and a clustering-based post-processing scheme. The proposed method accurately classifies various land cover (LC) types in multispectral satellite images, including Pastures, Other Built-Up Areas, Water Bodies, Urban Areas, Grasslands, Forest, Farmland, and Others. The post-processing scheme includes a spectral bag-of-words model and K-medoids clustering to refine the Deeplab v3+ outputs and correct possible errors. The simulation results indicate that combining the post-processing scheme with deep learning improves the Matthews correlation coefficient (MCC) by approximately 5.7% compared to the baseline method. Additionally, the proposed approach is robust to data imbalance cases and can dynamically update its codewords over different seasons. Finally, the proposed synergistic semantic segmentation method was compared with several state-of-the-art segmentation methods in satellite images of Italy’s Lake Garda (Lago di Garda) region. The results showed that the proposed method outperformed the best existing techniques by at least 6% in terms of MCC.

## 1. Introduction

Land cover classification (LCC) using remote sensing images aims to generate schematic maps from satellite and drone imagery [1]. This process involves creating a numerical representation based on available data for specific land cover (LC) types, such as forests, grasslands, pastures, water bodies, buildings, polluted areas, and mining zones [2,3]. By applying a computational classification method, the LC type in each different region of an image can be accurately identified. With the expansion of imaging and remote sensing drones and satellites, along with the increased accuracy and range of wavelengths acquired by sensors, LCC using remote sensing imagery has seen significant growth [4,5]. LCC has widespread applications across various fields, such as ecology, geography, climatology, and mapping [4,5]. Monitoring environmental changes using satellite imagery allows for detecting anomalies and changes, such as the degradation of natural resources [6]. This allows managers and planners to assess pollution and climate change with exceptional speed and precision. With the rapid growth of artificial intelligence, particularly in Computer Vision, supervised and semi-supervised methods for satellite image segmentation and classification have significantly improved in versatility and accuracy [7,8]. However, due to the complexity of specific patterns and the demand for highly detailed classifications, existing methods still require further refinement and enhancement [8].

Unlike traditional segmentation approaches, deep learning-based methods can identify more diverse areas and complex patterns. However, they require significantly more input data and larger training samples, more advanced hardware, and longer training times [5,9]. In the most recent studies, the combined use of expert knowledge in satellite image interpretation and artificial intelligence capabilities has improved the accuracy and generalizability of these methods [10,11]. Semantic segmentation, inspired by the functioning of the human mind, is one of the most accurate and advanced methods for segmenting satellite images. It leverages the capabilities of artificial intelligence to achieve more precise classifications. In a semantic segmentation, the input image is divided into multiple subsections, and each subsection is analyzed individually. The results are then processed, and, using a part-to-whole approach, the final segmentation of the entire image is achieved [4]. Semantic segmentation has shown highly satisfactory results across various fields, particularly satellite imagery. Given that modern sensors acquire a broader range of wavelengths compared to standard Red, Green, and Blue (RGB) images, using the entire frequency bands of satellite and drone sensors can enhance the segmentation quality and significantly improve the detection accuracy. In such scenarios, multispectral satellite images enhance the quality and efficiency of segmentation, particularly under varying weather conditions. For example, using near-infrared (NIR) bands helps distinguish vigorous vegetation, identify regions experiencing water stress, and monitor seasonal plant changes [12].

Various semantic LCC methods based on supervised deep learning techniques or semi-supervised approaches using transformers have been proposed [1,2,4,5]. However, based on our knowledge, no method has yet been introduced that combines expert knowledge with an intelligent approach for LCC synergistic semantic segmentation in a hybrid manner. In such cases, traditional methods and expert knowledge can correct some errors in the deep learning output, allowing the simultaneous benefit of both approaches. The present study proposes a hybrid approach combining a pre-trained Deeplab v3+ [13] segmentation network and a post-processing step using a dictionary containing spectral ensembles of various LC types for synergistic semantic segmentation. The innovations of the proposed method can be summarized as follows:

- Simultaneous use of the Deeplab v3+ model with a dictionary-based method for semantic segmentation;
- Building of a spectral dictionary for different LC types, which also covers seasonal changes;
- Hierarchical application of deep learning-based synergistic semantic segmentation, followed by result refinement using the dictionary-based method;
- Integration of deep learning and dictionary-driven methods to improve the learning speed and the output accuracy simultaneously.

This article is organized into five additional sections. Section 2 reviews the existing literature related to LCC. Section 3 explains the studied area and the used dataset in detail. Section 4 outlines the methodology and theoretical aspects employed in the proposed approach. Section 5 presents and discusses the obtained results. Finally, Section 6 concludes this article and suggests potential future research directions.

## 2. Literature Review

In recent years, the rapid development of artificial intelligence systems and the increasing availability of satellite imagery have significantly boosted progress in LCC. Some of the latest studies and advancements in this field focus on using multispectral satellite images. Levering et al. (2021) [14] proposed an approach combining LCC with landscape esthetic assessment using Sentinel-2 imagery across the United Kingdom. Their ScenicNet architecture employs a ResNet-50 [15] backbone for feature extraction, coupled with a semantic bottleneck that enables interpretable multi-task learning through class-specific modes. The semantic segmentation methodology leverages a multi-label classification approach rather than pixel-wise segmentation using 10 m resolution bands from the Sentinel-2 satellite. Baudoux et al. (2021) [16] introduced a framework for translating between different LC maps through a context-aware semantic segmentation approach that simultaneously handles spatial and nomenclature transitions. Their method employs an asymmetric U-Net architecture enhanced with positional encoding to capture local and global geographical context—a crucial consideration for large-scale LC mapping.

Walsh et al. (2021) [17] introduced a synergistic approach to land cover classification by employing a Resnet-50 Convolutional Neural Network (CNN) architecture for feature extraction and classification, adapted through transfer learning to serve as a segmentation algorithm. The segmentation process uses a U-Net architecture, which excels in pixel-level classification, which is essential for generating detailed land cover maps. The classifier was retrained on Sentinel-2 satellite imagery. Dabija et al. (2021) [18] conducted a comparative study on LCC using support vector machines (SVMs) and random forest (RF) algorithms, leveraging Sentinel-2 and Landsat 8 multispectral satellite images. The research used multi-temporal feature extraction to analyze seasonal variations and implemented pixel-based classification with iterative accuracy assessment for robust model evaluation. Baudoux et al. (2021) [19] proposed a novel map translation framework that directly infers CORINE land cover (CLC) maps from existing national-scale products, avoiding the need for new satellite imagery. Their approach leverages a CNN with asymmetrical architecture and positional encoding to harmonize spatial and semantic discrepancies between high-resolution source maps, mainly OSO, and the coarser CLC target. Rousset et al. (2021) [20] evaluated deep learning techniques for LCC using multispectral satellite imagery. The study used a custom dataset of five regions in New Caledonia, incorporating five LC classes with features derived from raw RGB and NIR bands. The study compared pixel-wise and semantic segmentation methodologies, employing CNNs, mainly DenseNet and DeepLabV3+, alongside a gradient-boosted decision tree classifier, XGBoost. DenseNet and DeepLabV3+ achieved the highest accuracy.

Martini et al. (2021) [21] proposed a novel methodology integrating domain-adversarial training with self-attention-based Transformer encoders to enhance LCC accuracy across geographic regions and leveraging multispectral, multi-temporal Sentinel-2 imagery. The study extracted temporal correlations using 10 spectral bands and 45 temporal steps. The model employs domain-adversarial neural networks (DANNs) to bridge domain discrepancies, with classification achieved via a Transformer encoder and multi-layer perceptron heads for LC prediction and domain alignment. Xie and Niculescu (2021) [22] investigated LCC changes over 11 years using SPOT-5 and Sentinel-2 satellite images. They analyzed deep learning and machine learning classifiers, including SVM, RF, and CNN, with the CNN achieving superior accuracy. Šćepanović et al. (2021) [23] explored semantic segmentation for wide-area LC mapping using Sentinel-1 C-band synthetic aperture radar (SAR) imagery, leveraging its resilience to cloud cover and low-light conditions. They used CORINE LC maps as reference data, focusing on five aggregated classes, and employed seven state-of-the-art segmentation architectures, including U-Net, DeepLabV3+, PSPNet, and FC-DenseNet, pre-trained on ImageNet and fine-tuned for SAR.

Yuan et al. (2022) [24] proposed SITS-Former, a pre-trained spatial–spectral–temporal representation model designed for LCC from Sentinel-2 satellite time series. The methodology employs a Transformer encoder backbone, pre-trained through a self-supervised learning task of missing-data imputation to capture high-level spatial and temporal dependencies. Sengan et al. (2022) [25] proposed a hybrid learning model, RAVNet, for efficient LCC using multispectral satellite imagery. The model integrates residual attention mechanisms with the VNet framework to blend low-level and high-level feature maps, enhancing spatial and contextual information extraction. Paris et al. (2022) [26] developed a scalable, high-performance, unsupervised system for producing high-resolution LC maps. Their methodology employs a tile-based, parallelizable approach using Sentinel-2 imagery. The feature extraction step incorporates robust spectral indices, while the classification step relies on an ensemble of SVMs with Gaussian radial basis functions. The segmentation step is achieved through K-means clustering to refine “weak” training sets extracted from coarse LC map units.

Zaabar et al. (2022) [27] suggested an integrated framework combining CNNs and object-based image analysis (OBIA) for LCC mapping in coastal areas. Using Sentinel-2 and Pléiades imagery, their methodology leverages CNNs to extract high-level spectral features through convolutional, pooling, and hidden layers, subsequently applying OBIA for segmentation and classification of LCC categories. The study also compared traditional machine learning classifiers, including RF and SVM, highlighting the superior accuracy of the OBIA-CNN integration. Efthimiou et al. (2022) [28] developed a high-resolution LCC approach to address the spatial and temporal limitations of the CORINE dataset. By integrating the land parcel identification system (LPIS) with multispectral Sentinel-2 imagery, the approach employs object-oriented segmentation and harmonization of datasets to enhance agricultural classification. Giffard-Roisin et al. (2022) [29] presented an innovative approach for LCC in the Alps using temporal coherence matrices derived from Sentinel-1 SAR data. The approach employs a one-year coherence matrix as input, capturing temporal and spatial patterns essential for segmentation. The features are extracted by treating the matrix as image-like data, enabling multi-scale texture analysis. The classification is performed using an SVM and CNN across six classes. Soni et al. (2022) [30] presented an urban LCC classification framework leveraging Sentinel-2 multispectral imagery to address challenges posed by high-density urbanization in South-West Delhi. Employing SVM, artificial neural networks (ANNs), and maximum likelihood classification (MLC) approaches, the study compared their performance using kappa coefficients and overall accuracy (OA) metrics.

Matcı and Avdan (2022) [31] proposed a methodology for the automatic labeling of LC classes using Sentinel-2 multispectral imagery, focusing on regions in Turkey and Greece. The classification spans five major categories, leveraging a pre-constructed spectral database alongside Corine LC data to validate and refine labels. This study highlights the potential of spectral-index-based models in addressing challenges in LCC, offering a scalable solution with enhanced spatial detail critical for ecological monitoring and resource management in remote sensing applications. Daniele la Cecilia et al. (2023) [32] introduced the open field and protected agriculture classifier (OPAC), a pixel-based model leveraging Sentinel-2 L2A imagery for LCC, addressing the unique challenges of mapping heterogeneous agricultural landscapes. Employing the RF algorithm, OPAC extracts features from a 13-dimensional vector of spectral bands to classify nine LC types.

Matei and Koßmann (2023) [33] introduced a robust Self-Supervised Learning (SSL) framework for addressing the challenges of season-invariant LCC using remote sensing data. The methodology leverages SeasoNet [34], comprising multispectral Sentinel-2 imagery with high-resolution segmentation labels, and employs MoCo-v2 for SSL pre-training with ResNet-50 and DeepLabV3 architectures. Feature extraction involves contrastive learning, incorporating novel seasonal augmentations and combinations with traditional artificial augmentations. Duarte and Fonte (2023) [35] proposed a framework to classify non-residential built-up areas by integrating national census data with Sentinel-2 satellite imagery through a supervised CNN segmentation model. The study employed census datasets combined with built-up data to automatically generate training masks, enabling segmentation using a modified U-Net architecture with densely connected layers to address class imbalances. Sentinel-2’s 10 m spatial resolution bands are used for feature extraction to differentiate residential and non-residential land uses.

Kramarczyk and Hejmanowska (2023) [36] employed a U-Net neural network architecture to classify Sentinel-2 multispectral satellite images for LCC in rural areas, addressing challenges in distinguishing agricultural and quarry land. The model leverages multi-temporal Sentinel-2 data to extract features across ten spectral bands, enabling detailed monitoring of LC transitions and soil conditions. Demir and Musaoglu (2023) [37] proposed a semantic segmentation framework leveraging deep learning for CORINE LCC using Sentinel-2 imagery. The methodology involves dataset pre-processing, U-Net architecture enhanced with ResNet50 and ResNet101 backbones, and transfer learning for robust feature extraction. This approach employs multi-temporal Sentinel-2 data, including RGB and NRG bands, facilitating seasonal variability assessments. Zamanoglu et al. (2023) [38] suggested a hybrid semantic segmentation approach combining DeepLabV3 and ResNet34 architectures for LCC using the LandCover AI dataset. The model leverages ResNet34 for robust feature extraction and employs DeepLabV3 to handle multi-scale contextual information. Cecili et al. (2023) [39] explored CNNs for LC mapping, leveraging Sentinel-2 multispectral imagery. The study evaluated DenseNet121, ResNet50, and VGG16 models using single-date and multi-temporal datasets, ultimately identifying VGG16 as the most effective classifier.

Tzepkenlis et al. (2023) [40] presented a novel approach to LCC using a modified U-TAE model for Sentinel imagery composites processed via Google Earth Engine. Their methodology simplifies the input data by employing temporal median composites of Sentinel-1, Sentinel-2, and ALOS elevation data, reducing noise from atmospheric effects. Feature extraction leverages a channel attention mechanism within the U-TAE model, diverging from traditional temporal attention strategies. Cuypers et al. (2023) [41] proposed an integrative approach for LCC mapping by leveraging very high-resolution (VHR) optical imagery and multi-temporal Sentinel-2 satellite data within a geographic object-based image analysis (GEOBIA) framework. The methodology incorporated RF classifiers, augmented with simple non-iterative clustering (SNIC) for segmentation, and extracted features such as gray-level co-occurrence matrix (GLCM) textures and temporal indices, such as the phase and amplitude of spectral indices. Arrechea-Castillo et al. (2023) [42] proposed a robust, computationally efficient approach for multi-class LCC classification using Sentinel-2 imagery and a simplified CNN based on the LeNet architecture. Their model used 27 features derived from pre-processed spectral bands, a digital elevation model (DEM) and 16 radiometric indices. Fagua et al. (2023) [43] developed a high-resolution LCC framework tailored to tropical regions using temporal metrics derived from Sentinel-1 SAR and Sentinel-2 multispectral data. The study integrated SAR backscatter coefficients and multispectral indices with visual pixel classifications and field survey data. Five machine learning classifiers were evaluated, with RF achieving the best performance.

Gharbia (2023) [44] introduced an automated framework for extracting water regions using Faster R-CNN, a region-based CNN designed for object detection. This method integrates CNN-based feature extraction with a region proposal network (RPN) to achieve precise classification and localization of water features. The approach was evaluated using Sentinel-2 and Landsat-8 (OLI) datasets, with Sentinel-2 leading to the highest accuracy. Kavran et al. (2023) [45] introduced a spatiotemporal approach for LCC using multispectral Sentinel-2 satellite images processed through a graph neural network (GNN). The methodology integrated superpixel segmentation with graph-based representation, where segmented land regions across sequential images were modeled as directed graphs. Feature extraction was conducted using EfficientNetV2-S, while node classification relied on the GraphSAGE algorithm with LSTM-based aggregation. Carneiro et al. (2023) [46] proposed a transfer learning framework using small 3D CNNs for LCC. Their method used semantic segmentation with a slide-window approach, using pre-trained models fine-tuned on Sentinel-2 imagery, with bands at 10 m and 20 m resolutions. Feature extraction incorporated spectral–spatial characteristics via small CNNs and ResNext50 as the backbone for specific segmentation tasks.

Çelik and Gazioğlu (2024) [47] employed a modified VGG16 CNN using transfer learning for the semantic segmentation of coastal LC using Sentinel-2A multispectral imagery. Their methodology used Google Earth Engine for large-scale data pre-processing and incorporated spectral band combinations, notably emphasizing the NIR band, to enhance classification accuracy across five coastal classes. Feature extraction relied on the fine-tuned later layers of VGG16, while classification employed the CNN’s architecture with adjustments for improved generalizability. Pešek et al. (2024) [48] proposed a CNN-based framework for semantic segmentation of urban green areas using Sentinel-2 multispectral imagery. The study evaluated four CNN architectures, FCN, U-Net, SegNet, and DeepLabv3+, and compared them to an RF baseline. This work underscores CNNs’ potential in addressing LCC challenges, particularly for urban environments with limited high-resolution datasets. Perez-Guerra et al. (2024) [49] explored deep learning-based semantic segmentation techniques for LCC using Sentinel-2 multispectral images. The study employed U-Net, U-Net++, and PSPNet architectures, integrating feature extraction through ResNet and ResNeXt backbones, pre-trained on ImageNet. Vo Quang et al. (2024) [50] used CNNs to identify degraded forests using Sentinel-2 multispectral imagery. The study used U-Net, SegNet, and ResNet-UNet models, with U-Net demonstrating superior performance.

Kalaivani et al. (2024) [51] presented a comprehensive approach to LC segmentation using a blend of state-of-the-art deep learning architectures, including U-Net++, DeepLabV3+, InceptionV4, MobileNetV2, and ResNet152. While the research underscores the effectiveness of combining high-performing models for segmentation, its reliance on existing datasets may limit adaptability to unexplored geographic regions, reflecting broader challenges in scalable LCC from multispectral satellite imagery. Marko Pavlovic et al. (2024) [52] proposed a two-stage deep learning pipeline for estimating soil organic carbon (SOC) using Sentinel-2 satellite imagery, emphasizing LCC as a precursor to SOC prediction. The methodology employs the U-Net architecture for image segmentation to extract spatial features from multispectral images, subsequently using these as input for machine learning models such as Extremely Randomized Trees, which achieved superior performance. Suraj Sawant and Ghosh (2024) [53] used a tailored semantic segmentation approach to address the challenges of LCC using Sentinel-2 imagery. Their methodology involved training five state-of-the-art deep CNNs, including UNet, FPN, and LinkNet architectures optimized for pixel-wise classification of seven LCC classes.

Sharma et al. (2024) [54] introduced Sen4Map, a benchmark dataset built for detailed land cover mapping using Sentinel-2 satellite data. Feature extraction incorporates Sentinel-2 bands at 10 m and 20 m resolutions, excluding bands primarily used for atmospheric corrections. Four classifiers, including RF and temporal vision transformers, were benchmarked for broad land cover categorization and detailed crop classification, emphasizing temporal harmonization. Lasko et al. (2024) [55] proposed a scalable LCC methodology for Sentinel-2 imagery across seven diverse global sites. The framework integrates binary masks derived from spectral, textural, and ancillary geospatial data layers and optimizes thresholds regionally and globally to generate nine-class, six-class, and five-class models. The segmentation approach combined adaptive thresholding with decision functions, ensuring compatibility across heterogeneous landscapes. Some studies used vision transformers [24,56] and self-supervised learning approaches [33,34] to improve LCC methods. However, these methods did not use traditional post-processing techniques to correct errors in segmentation output.

Based on the reviewed studies, while deep learning and some machine learning methods have shown good performance in LCC, there are still various challenges. One of the main challenges is the need to have a large amount of training data to improve accuracy and generalizability. The aforementioned studies are all trained on local and regional data, restricting the models’ generalizability. Additionally, they must be trained for various times of the year to operate independently of temporal factors in vegetation or forest classification. In some applications, such as mining, spectral information databases are available; however, spectral data for other types of coverage remain quite limited. This study introduces a combined deep learning and multispectral analysis approach for LCC.

In the current study, a deep learning LCC model is used for segmentation, and then a K-medoids post-processing step is used to improve the segmentation result. The post-processing step uses an assumption of continuity in adjacent regions to fix errors. By combining the strengths of both deep learning and K-medoids, the proposed approach aims to achieve better performance. Leveraging transfer learning significantly reduces the number of samples required for training the deep learning model. Additionally, dictionary-based multispectral analysis is incorporated to enhance the accuracy of the synergistic semantic segmentation, so the proposed method effectively addresses the limitations of previous techniques to a notable extent.

## 3. Studied Area and Dataset

### 3.1. Studied Area: Lake Garda

**Figure 1:**
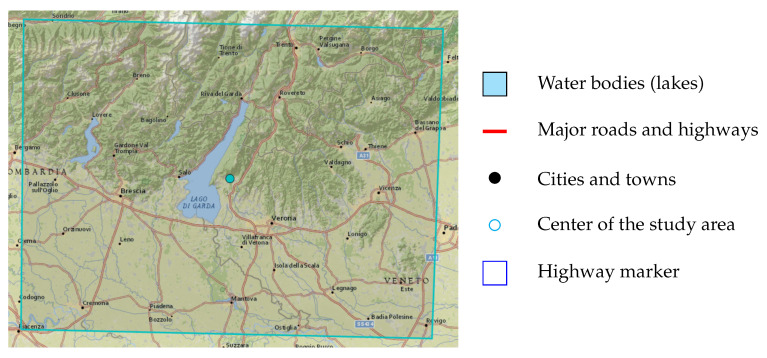
Map of Lago di Garda.

Lake Garda (Lago di Garda) is the largest lake in Italy, situated in Northern Italy between Brescia in the Lombardy region and Verona in the Veneto region. Figure 1 shows a map of the studied area. The Lake Garda area includes diverse water bodies, farmlands, various orchards, urban areas, pastures, and forests. Due to its geographical and climatic diversity, this region is considered an ideal location for LC analysis. In addition to its natural features, the area has a well-developed infrastructure, including transportation networks and expanding urban areas. This diversity presents significant challenges for accurately classifying LC types [4].

**Figure 2:**
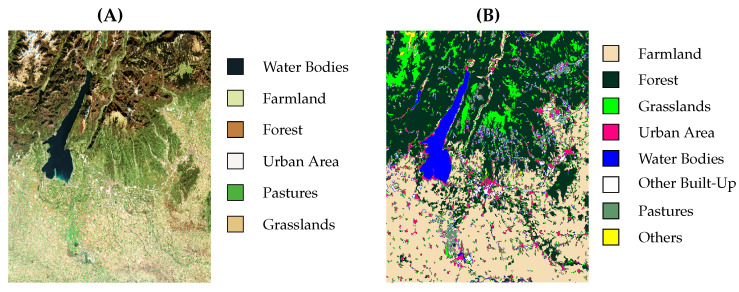
True colour (**A**) and land classification (**B**) images of the studied area.

The current study uses Sentinel-2 satellite data obtained from Copernicus as the Earth observation component of the European Union’s Space program. The images were acquired during the four seasons of 2024 to minimize the impact of seasonal LC variations. Figure 2A illustrates the RGB band image of the studied area taken by Sentinel-2. Figure 2B shows the LC of the same area taken from the CORINE Land Cover 2018 dataset. The Sentinel-2 satellites are multispectral Earth observation satellites consisting of different bands. Two Sentinel satellites were launched as part of the European Union’s Earth Observation Program in 2015 and 2017. The primary objectives of these satellites include monitoring the Earth’s surface, supporting environmental applications, and studying climate change. The Sentinel-2A satellite, whose spectral bands are listed in Table 1, can acquire images of a 290 km wide area of the Earth’s surface and revisit each location every five days. Other notable features of this satellite include spatial resolutions of 10, 20, and 60 m.

The Sentinel-2 bands 1 to 9 and 11 and 12 were used for this study. The other band, specifically designed for detecting cloud and atmospheric particles, was not considered relevant for the segmentation process.

### 3.2. CORINE Land Cover 2018 Dataset

The CORINE Land Cover 2018 dataset was used for training and validating the proposed model. This dataset provides LCC across Europe with 44 distinct classes and is widely recognized as a reliable standard for training and evaluating the performance of segmentation models [4,57,58,59]. The key features of this dataset include thematic accuracy and spatial resolution, which are indicated in Table 2. The dimension of the CORINE LC image of the studied area is 5490 × 5490.

The selected area was analyzed across eight LC classes: Pastures, Water Bodies, Grasslands, Urban Areas, Farm Land, Forest, Other Built-Up, and Others. The samples were selected using QGIS tools and the Semi-automatic Classification Plugin (SCP). Using the graphical user interface, the Sentinel-2 data were manually downloaded from the Copernicus website. The details of the selected geographic area include a longitude range of [9.727, 11.814] E and a latitude range of [45.023, 46.178] N. The specifications of atmospheric and geometric corrections applied to downloaded images are listed in Table 3.

## 4. Proposed Method

**Figure 3:**
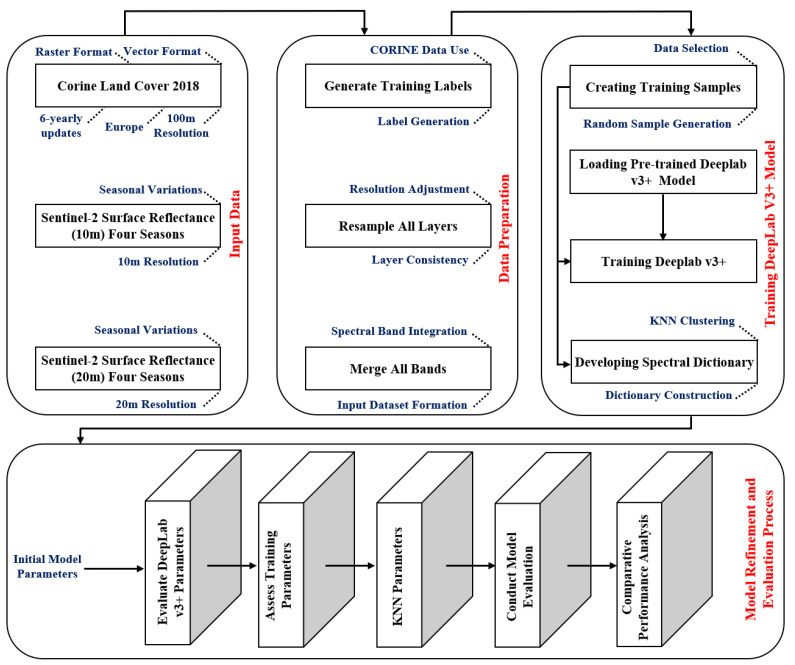
Block diagram of the proposed method.

The block diagram of the proposed method is presented in Figure 3, which consists of four main stages: data input, data preparation, deep learning, and post-processing/classification. The first two stages, data input and preparation, ensure a suitable input for the deep learning and post-processing steps, which require training. In the first step, data related to the study area, including the various bands of the Sentinel-2 satellite and the Corine Land Cover 2018 dataset, are read. In the data preparation step, Sentinel-2 satellite images and the Corine LC image are resized to match each other. A multispectral 12-layer image is created using Sentinel-2 resized, which includes all the spectral bands listed in Table 1. During the training phase, 224 × 224 × 12 patches are generated from the multispectral image created in the data preparation step to train and evaluate the method’s performance. These patches are used in two blocks: training the deep neural network and extracting the dictionary codewords blocks. For the dictionary and the deep learning model to perform well across all four seasons of the year, the samples for deep learning and the dictionary must be selected from all seasons.

### 4.1. Deep Learning Model

**Figure 4:**
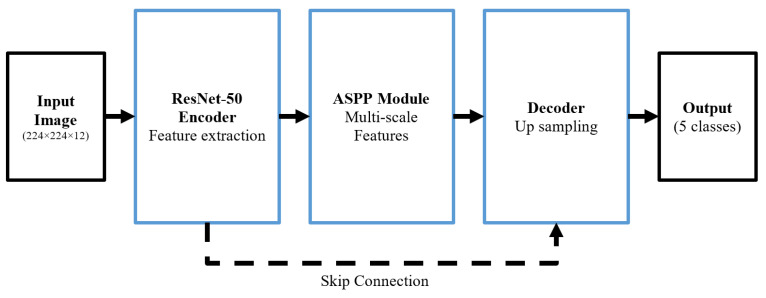
Block diagram of the DeepLabv3+ model.

The DeepLabv3+ architecture [61] is recognized as one of the most powerful models for semantic image segmentation [13,62,63]. By leveraging advanced deep learning techniques, it can extract complex semantic features from multispectral images. This architecture combines several approaches, such as Atrous Convolutions and multi-scale attention mechanisms, to achieve high performance in identifying and classifying Earth’s surface features. The DeepLabv3+ architecture combines the feature extraction capabilities of ResNet-50 with advanced mechanisms such as atrous spatial pyramid pooling (ASPP) and a decoder [64]. Leveraging innovative techniques like shortcut connections and an adaptive decoder effectively addresses key challenges in processing multispectral satellite imagery, including the scale diversity of features, spectral complexities, and significant variations in imaging conditions. As illustrated in Figure 4, the DeepLabv3+ architecture is composed of three main components: ResNet-50, the ASPP module, and the decoder [61,65,66].

The DeepLabv3+ model takes Sentinel multispectral satellite images with dimensions of 224 × 224 × 12 as input.

- ResNet-50 Encoder
  In the initial stage of ResNet-50 [15], a 7×7 convolutional layer with 64 filters, combined with a 3×3 MaxPooling operation, reduces the spatial dimensions while extracting low-level features such as edges, textures, and spectral patterns. After that, ResNet-50 processes the reduced input through four sequential stages:
  – Stage 1: Three residual blocks with a depth of 256 to extract basic features;
  – Stage 2: Four residual blocks with a depth of 512 to develop intermediate features;
  – Stage 3: Six residual blocks with a depth of 1024 to extract complex features;
  – Stage 4: Three residual blocks with a depth of 2048 to capture high-level semantic features.

**Figure 5:**
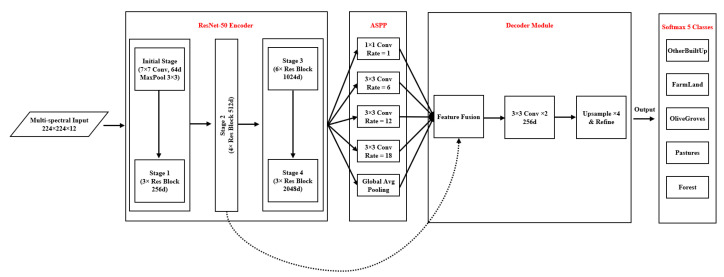
Blocks of the DeepLabv3+ model in detail.

Figure 5 shows details of the ResNet-50 blocks in the DeepLabv3+ structure.

- Skip Connection and Detail Preservation Mechanism
  In the DeepLabv3+ architecture, the skip connection [61,65,66] is a key component in addressing two significant challenges in deep neural networks.
- First challenge:
  The gradual loss of spatial information and image details as data move from the early layers to the deeper ones. This loss occurs due to convolution and down-sampling operations.
- Second challenge:
  Related to the fact that while deeper layers of the network, such as Stage 4, are effective at extracting high-level semantic features, they reduce the spatial details of the data.

To overcome these challenges, a direct connection is established between Stage 2 of the encoder and the Feature Fusion section of the decoder. This connection, represented by a dotted line in the flowchart (Figure 5), enables the direct transfer of high-resolution and spatial information from the intermediate layers to the decoder. The advantages of the skip connection are as follows:

- Preservation of edge details for detecting boundaries between LC classes.
- Improved spatial accuracy in detecting small areas.
- Intelligent combination of low-level and high-level features.
- ASPP Module—This module consists of three parallel pathways:
  – 1 × 1 convolution with an Atrous rate of 1 (one) for capturing local features.
  – Three 3 × 3 convolution layers with Atrous rates of 6, 12 and 18 to cover different receptive fields.
  – Global Average Pooling branch for understanding the overall context of the image.

This multi-scale module allows the model to identify land cover features at various scales effectively [56]. The decoder architecture consists of three main stages:

- Feature fusion to combine precise spatial information with semantic data.
- Two 3 × 3 convolution layers with 256 filters to refine the fused features.
- A 4× up-sampling step to restore the image to its original dimensions.

Finally, a Softmax layer classifies the output into eight LC classes: Pastures, Water Bodies, Grasslands, Urban Areas, Farm Land, Forest, Other Built-Up, and Others. This architecture achieves a balanced trade-off between spatial accuracy and semantic depth by intelligently integrating various techniques. Its ability to process multi-scale data while preserving spatial details makes the model highly effective for synergistic semantic LCC using satellite imagery.

### 4.2. K-Medoids

Selecting a robust and efficient algorithm is essential in clustering large and complex spectral information, such as the multispectral satellite images examined in this study. The K-medoids algorithm is a clustering method similar in structure to K-means but that uses actual data points as cluster representatives instead of the mean. K-medoids provides more excellent resistance to noise and outliers than K-means. This characteristic is especially important for inherently noisy data, such as multispectral remote sensing data.

Here, *M* spectral bands of pixels are considered as a set of M-dimensional points $\left\{x_{1},x_{2},\ldots ,x_{n}\right\}$. The goal of the K-medoids algorithm is to find a set of *k* medoids $\left\{m_{1},m_{2},\ldots ,m_{k}\right\}$ such that the total distance between the data points and their nearest medoid is minimized, or equivalently, the similarity between the selected medoids and the data points is maximized. Mathematically, this can be expressed as:

(1) $$\min_{M}\sum_{i=1}^{n}\min_{j=1}^{k}d\left(x_{i},m_{j}\right),$$

where $d(x_{i},m_{j})$ is a distance metric, such as the Euclidean or Manhattan distance, and $m_{j}$ represents one of the actual data points, not a computed value. If a similarity metric instead of a distance metric is used, the Min(minimums) in Equation (1) should be replaced with the maximum. $m_{j}$ ensures the algorithm is more robust against outliers.

The K-medoids algorithm consists of three main steps:

- Initial Selection of Medoids:
  Medoids are initially selected either randomly or based on statistical criteria such as data density in the *M*-dimensional data space. The medoids are chosen directly from the available data points. In the study by Park and Jun [67], selecting the initial medoids based on the smallest computed distance ratio among data points is suggested to accelerate convergence.
- Assigning Data to Medoids:
  Each data point is assigned to the nearest medoid based on a defined distance metric. This step clusters the data points around their respective medoids.
- Updating Medoids:
  For each cluster, the point with the smallest total distance to all other points in the same cluster is chosen as the new medoid. This ensures that the new medoid best represents the cluster members.

This process is repeated until the medoids either stop changing or the changes become negligible. The extracted medoids can be used in images acquired by other satellite sensors according with the Sentinel 2 spectral bands or from different geographic regions. This possibility contributes significantly to the generalization of the proposed method.

### 4.3. Relationship with Multispectral Satellite Images

Multispectral satellite images consist of numerous spectral bands, where each pixel in an image has a reflectance value for every band. These data can be represented as multidimensional vectors. The K-medoids algorithm can effectively cluster these vectors and associate each cluster with a specific LC type. For example, one medoid of pixels might represent forests, while another could correspond to agricultural areas. In this study, medoids can also include seasonal information about natural features, as the spectral characteristics of natural covers change during different times of the year.

### 4.4. Advantages of Using K-Medoids in Synergistic Semantic Segmentation

- Resistance to Noise:
  Since medoids are real data points, the algorithm becomes more resistant to noise and outliers.
- Flexibility in Distance Metrics:
  K-medoids allows for the use of various distance metrics, making them suitable for multispectral data with different scales.
- Application in Hybrid Models:
  This algorithm can be used as a refinement step after deep learning models in synergistic semantic segmentation, resulting in more accurate segmentations.
- Improved Convergence Speed:
  The K-medoids algorithm proposed by Park and Jun [67] demonstrates faster convergence, particularly with large datasets, as in the present study, compared to similar clustering algorithms.
  The computational complexity of traditional K-medoids methods like PAM is $O(k{\left(n-k\right)}^{2})$, which is computationally expensive for large datasets. However, Park and Jun [67] reduced this complexity to O(nk) by using a distance matrix and minimizing calculations.

## 5. Results

In this section, the evaluation metrics for the proposed synergistic semantic segmentation method are defined, and the influence of different learning parameters on the model’s output is analyzed. A series of simulations were performed on the study area to determine the unknown parameters of the deep learning and K-medoids approaches. Once the optimal parameters were identified, the proposed method was evaluated and compared with several of the latest deep learning-based semantic classification approaches. The results highlight the proposed approach’s efficiency in addressing semantic segmentation challenges and demonstrate its competitive performance against state-of-the-art methods.

### 5.1. Evaluation Metrics

This study conducted evaluations based on the confusion matrix generated from the test data and various common metrics derived from it. The confusion matrix encompasses all possible outcomes produced by the LCC model. From this matrix, four key values are extracted for each class:

- True Positive (TP): pixels that are correctly classified as belonging to the respective class;
- False Positive (FP): pixels that are incorrectly assigned to the respective class;
- False Negative (FN): pixels that are incorrectly excluded from the respective class;
- True Negative (TN): pixels that are correctly classified as belonging to other classes.

In a multi-class classifier, the true negative value for each class is the sum of all elements in the confusion matrix that are not located in that class’s corresponding row and column. The sum of these four values for any class always equals the total number of samples in the matrix. These values provide the foundation for a detailed evaluation of the proposed model’s performance across various classes and methods. Using these key values, the following metrics were calculated and used for evaluation in this study: precision, recall, F1-score, Overall accuracy, Matthews correlation coefficient, and the weighted average of metrics.

#### 5.1.1. Precision

Precision is an important metric for assessing the accuracy of predictions and represents the proportion of correctly predicted samples to the total predicted samples for a specific class. The formula for precision is as follows:

(2) $$\mathrm{Precision}=\frac{TP}{TP+FP}.$$

This metric, which depends on the number of FP, is particularly critical for classes where FP errors can lead to significant consequences. For instance, misclassifying other classes as water can result in a significant upward bias in estimating water-covered areas in the segmentation of Water Bodies. This bias can cause substantial issues in agriculture planning based on surface water resources and urban water management.

#### 5.1.2. Recall

Recall is a metric that measures a model’s ability to identify true instances correctly and emphasizes the importance of minimizing missed predictions in real data. It is defined as follows:

(3) $$\mathrm{Recall}=\frac{TP}{TP+FN}.$$

For example, in identifying Forests, a recall value close to one ensures that all forest regions in the image are correctly identified. Missing Real Data (FN) can lead to inaccurate estimates in forest cover analysis and environmental management, highlighting the importance of this metric in applications where completeness is critical.

#### 5.1.3. F1-Score

F1-score is a composite metric that measures the balance between precision and recall, making it particularly useful in cases where there is a trade-off between these two metrics. The formula for F1-score is:

(4) $$F1=\frac{2\times TP}{(2\times TP)+FP+FN}.$$

This metric is especially valuable in classifications such as Pastures and Farmland, where similar spectral and spatial features can lead to overlapping characteristics. By combining precision and recall into a single value, F1-score provides a more accurate evaluation of the model’s performance in such scenarios.

#### 5.1.4. Overall Accuracy

Overall accuracy (OA) represents the percentage of pixels correctly classified by the model. It is defined as:

(5) $$OA=\frac{TP+TN}{TP+FP+TN+FN}.$$

While this metric provides a simple general overview of the model’s performance, it can be misleading in imbalanced datasets. For instance, if the ‘other’ class constitutes only a small portion of the data, the model might achieve a high OA without performing well in this specific class. This limitation underscores the importance of complementing OA with other metrics, particularly in cases of class imbalance.

#### 5.1.5. Matthews Correlation Coefficient

The Matthews correlation coefficient (MCC) is a robust metric that evaluates the performance of a model, even in cases where class distributions are imbalanced, and is defined as:

(6) $$MCC=\frac{\left(TP\times TN)-(FP\times FN\right)}{\sqrt{\left(TP+FP)\times (TP+FN)\times (TN+FP)\times (TN+FN\right)}}.$$

This metric is particularly useful for classes like Others, which often contain sparse and imbalanced data. By accounting for all elements of the confusion matrix, the MCC provides a comprehensive evaluation of the model’s performance, making it ideal for scenarios with unbalanced class distributions.

#### 5.1.6. Weighted Average of Metrics: Emphasizing More Significant Classes

The weighted average of metrics highlights the importance of specific classes. This approach calculates the average by incorporating the number of samples for each class, ensuring that metrics for more significant or abundant classes are appropriately emphasized. The formula is:

(7) $$\mathrm{Weighted}\mathrm{Average}=\sum_{i=1}^{K}w_{i}\cdot {\mathrm{Metric}}_{i},$$

where $w_{i}$ represents the weight of class *i*, typically the ratio of the number of samples in class *i* to the total samples, $Metric_{i}$ is the evaluation metric, such as recall, precision, or F1, for class *i*, and *K* is the total number of classes. By applying this method, the evaluation results better reflect the importance of each class, especially in datasets with unbalanced distributions.

### 5.2. Model Parameter Evaluation

To train the synergistic semantic segmentation model, 49,439 patches of size 224 × 224 × 12 were extracted from the studied area to create training, testing, and validation sets. Out of this total, 60% (29,663 patches) were allocated for training, 20% (9888 patches) for testing, and 20% (9888 patches) for validation. The allocated patches for training, testing, and validation were selected randomly once and were used across all stages, including training, model parameter selection, and comparison with other methods. The distribution of training samples for each class in the CORINE LC image of the studied area is provided in Table 4, offering insights into the class-specific representation within the dataset. The deep learning model training was performed on an Nvidia DGX workstation with 128 GB RAM (four 32 GB Nvidia Tesla V100 GPUs) and 20,480 CUDA cores. An HP Z1 Tower G5 system was employed for initial pre-processing and patch generation, featuring 16 GB of RAM, a 512 GB SSD, an Nvidia 2070 Gaming GPU, and an Intel i7-9700 CPU. Training and validation data were used simultaneously to prevent overfitting during the DeepLab v3+ deep learning network training. Key parameters, including initialLearningRate, maxEpochs, and minibatchSize, were analyzed to assess their influence on system performance. These parameters were tested across ranges of initialLearningRate [0.0001–0.1], maxEpochs [20–500] and minibatchSize [16–256]. The coordinate descent scheme was used as an optimization algorithm for finding hyperparameters. Coordinate descent is based on the idea that minimizing a multivariable function can be achieved by minimizing it along one direction (variable) at a time. The cost function of the optimization algorithm was the maximization classification accuracy. Memory usage and training duration were also considered as secondary optimization criteria. The final values selected for training DeepLab v3+ are summarized in Table 5. The confusion matrix of the optimized DeepLab v3+ model on the test data is presented in Table 6. The OA in this step was 83.62%.

The K-medoids method involves three critical parameters: the distance or similarity metric, the number of medoids (or centers), and the number of input vectors in a single execution. All the three parameters influence the memory consumption and runtime of the method, while the distance metric and the number of medoids directly impact the system’s performance. The current study employs seven metrics to evaluate the similarity/distance between spectral vectors belonging to a class. Each vector comprises 12 spectral values corresponding to 12 frequency bands (N = 12). The selected distance metrics include the Squared Euclidean distance, Standardized Euclidean distance, City Block distance, Chebyshev distance, Cosine similarity, and Correlation distance. These metrics, described in Table 7, play a vital role in determining the clustering efficiency and the quality of the extracted medoids.

In the formulas presented in Table 7, $x_{i}$ and $y_{i}$ represent the $i_{th}$ spectral values in two vectors from the same class; $Z_{x_{i}}$ refers to the standardized variable, calculated using $Z_{x_{i}}=\frac{x_{i}-\mu_{i}}{\sigma_{i}}$, where $\mu_{i}$ and $\sigma_{i}$ are the mean and standard deviation of the $i_{th}$ spectral band in the corresponding cluster. For metrics such as City Block and Chebyshev, $\left|x_{i}-y_{i}\right|$ denotes the absolute difference between the two values. In the Correlation metric, $\mu_{x}$ and $\mu_{y}$ represent the mean values of vectors *x* and *y*, respectively. The Squared Euclidean distance, a fundamental metric, calculates the sum of squared differences between corresponding elements of the two vectors. It is highly sensitive to large deviations and exhibits faster convergence during clustering. Conversely, the Standardized Euclidean distance normalizes variables by accounting for their mean and standard deviation, making it more robust to differing scales but slower in convergence. The City Block distance, or the Manhattan distance, computes the sum of absolute differences between vector elements, offering a straightforward and interpretable measure. On the other hand, the Chebyshev distance considers only the maximum absolute difference, focusing on the most significant deviation between corresponding elements of the vectors. Cosine similarity measures the cosine of the angle between two vectors, emphasizing their directional similarity over magnitude. Meanwhile, the Correlation distance evaluates the linear relationship between vectors by centring them at their respective means and standard deviations, making it particularly effective in identifying patterns across spectral values.

Optimization was crucial given the extensive samples (pixels) used in this study, which comprised over 100 million data points in some classes. The input size was limited to 1 (one) million vectors per iteration to balance memory consumption and computational efficiency. The number of medoids was changed from 5 to 100, and it was found that accuracy improved until the number reached 50; beyond that, additional medoids did not significantly increase accuracy (see Table 8). Based on these results, 50 as the number of medoids was chosen as the optimal value. The medoids were refined using the K-medoids algorithm to handle classes with more data, such as Forest and Farmland. For classes with stable spectral characteristics across seasons, 50 final medoids were extracted. Conversely, for classes like Pastures, Grasslands, Forest, and Farmland, where spectral values vary with seasonal changes, 50 medoids were computed per season, resulting in 200 medoids per class. Each medoid, or “code word”, is represented as a 12-element spectral vector. The results highlight that the Squared and Standardized Euclidean distances are more accurate than others. Between these two metrics, Squared Euclidean demonstrated superior convergence speed compared to the Standardized Euclidean distance, making it a preferable choice for this application. Additionally, carefully adjusting input sizes and iterative refinement of medoids ensured a balance between computational feasibility and clustering accuracy, producing robust medoid representations for further analysis.

### 5.3. Post-Classification Refinement Using the Designed Dictionary

In the proposed synergistic semantic segmentation method, the designed dictionary is used after the deep learning-based classification step to refine the classification results. The refinement process involves analyzing each pixel and its eight neighbouring pixels. If a pixel and all its eight neighbours belong to the same class, the pixel is assumed to be correctly classified and excluded from further processing. This approach avoids unnecessary computations for pixels already deemed reliable. However, since changes to a single pixel may affect the classification of its neighbouring pixels, which are candidates for further processing, the refinement is conducted once across the entire image. If pixels with at least one neighbouring pixel belonging to a different class, i.e., non-homogeneous eight-neighborhood, are found, the refinement process is applied. Specifically, the class of the medoid closest to the spectral characteristics of a non-homogeneous eight-neighborhood pixel is assigned as the corrected class.

The final results of this scheme, which effectively refines deep learning predictions using dictionary-based corrections, are summarized in Table 9. This hybrid method ensures improved classification accuracy by leveraging the high-level abstraction capabilities of deep learning and the robust representation of medoids in the dictionary. As an initial evaluation, the OA of the DeepLab v3+ network was 83.62%. However, after applying the dictionary-based post-processing, this value improved to 88.4%. The results improved across all classes, meaning FP and FN decreased while TP and TN increased.

An error analysis highlights the proposed method’s advantages in specific land cover classes that are frequently misclassified. In Table 10 top and bottom, the confusion matrix of recall for both the baseline and the proposed method in the test set is presented. The presented errors show a random pattern for the baseline method (Table 10 top). After applying the K-medoids post-processing step (Table 10 bottom), a clear pattern emerges: the highest error in each class is linked to a similar class. For example, the most frequent misclassification for “Pastures” is with “Grasslands”, which are very similar cases. Another trend can be perceived for the “Forest” and “Farmland” categories. This shows that the proposed method greatly reduces the random errors, and the most remaining errors occur only between very similar classes. To reduce these remaining errors, a hyperspectral image with more spectral bands can be used.

### 5.4. Comparison with Other Methods

To evaluate the efficiency of the proposed method, the results obtained were compared with those of some of the recent works used in LCC. The selected models were based on [53]. Five hybrid networks were compared with the results from DeepLab v3+ and the final proposed approach. The chosen architectures include UNet [68], FPN [69], and LinkNet [70], all of which have been effectively used in semantic segmentation and have demonstrated promising performance. Additionally, backbone networks such as ResNet [15], DenseNet [71], VGG [72], and MobileNetV2 [73] were used. These backbones, pre-trained on the ILSVRC ImageNet 2012 dataset [74], were combined with the aforementioned architectures in Sawant and Ghosh [53]. Table 11 details the proposed method, the compared architectures, and their respective backbones. The proposed method is based on the DeepLab v3+ architecture and backbone. The networks were retrained based on the study problem and selected classes. All implementation details and the data used for training, validation, and testing followed the same approach as that used with the DeepLab v3+ network. The assumptions outlined in [53] were applied when additional parameters were required in the network structures.

**Figure 6:**
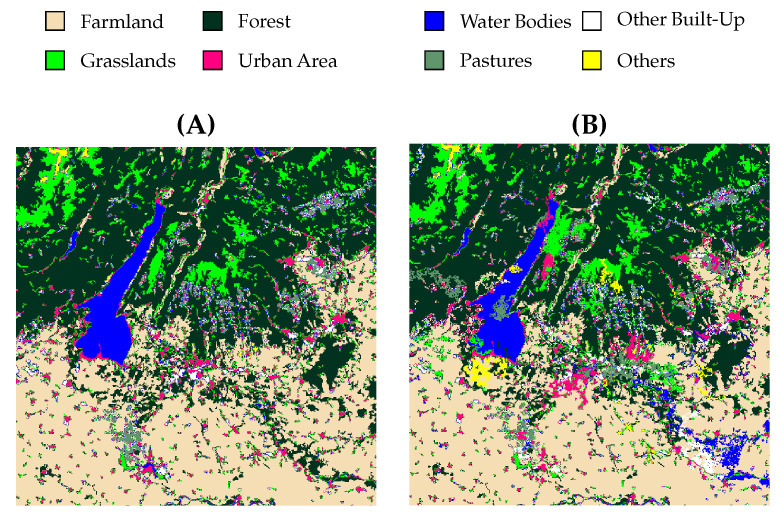

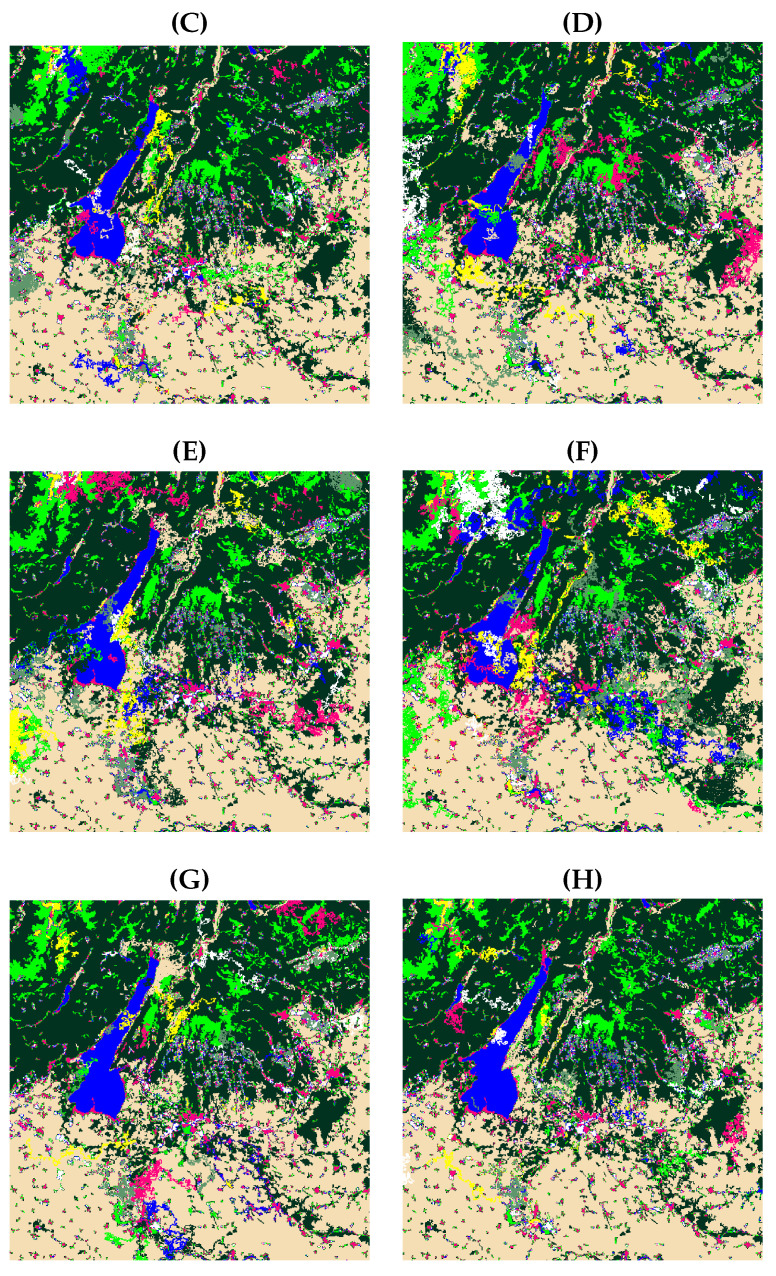
The LCC results of the proposed method alongside the comparison methods, including the ground truth: (**A**) ground truth, (**B**) UNet-ResNet50 [53], (**C**) UNet-ResNet152 [53], (**D**) UNet-DenseNet169 [53], (**E**) FPN-VGG16 [53], (**F**) LinkNet-MobileNetV2 [53], (**G**) Deeplab v3+ [13], and (**H**) proposed method.

Figure 6 shows the segmentation results of the proposed method alongside the comparison methods, including the ground truth. As can be seen, the proposed method has fewer errors than the others. The OA results of each LCC method are indicated in Table 11.

Based on the results presented in Table 11, the proposed approach demonstrates superior OA compared to the other networks. As an essential parameter, deep learning methods for satellite image segmentation always involve a trade-off between the computational complexity and the model’s accuracy and generalization. Lighter networks, such as MobileNet-based UNets, require less data and can run on simpler hardware, but they often deliver lower accuracy and generalization on a large scale. More complex networks typically need powerful servers to train and fine-tune on specific regions using transfer learning. Table 11 shows each network’s total and trainable parameters. Considering the number of trainable parameters, the proposed method is not very complex and can be trained on a desktop system, including a GeForce RTX 30- or 40-series graphics card. If lower-end hardware is preferred, there are two options: reduce the geographic area and the number of training samples, use a local server/cloud computing for training, and then run the trained model on simpler hardware. For instance, the trained model can run on a GeForce RTX 10-series graphics card. To provide a clearer understanding of the performance of the proposed method and the improvements achieved after applying post-processing, Table 12 presents the confusion matrices for the various architectures. Additionally, Table 13 calculates and compares the evaluation metrics for each class based on the results derived from the confusion matrices.

The results in Table 12 provide an explicit demonstration of the strengths of the proposed method compared to existing approaches. All architectures performed relatively poorly for classes with significantly fewer samples relative to the entire dataset. However, even in these cases, the proposed method obtained more robust results than the other approaches. The closest performance to the proposed method was observed with the UNet-ResNet152 network, though in most cases, there were significant differences favouring the proposed approach, particularly after applying the post-processing step. For instance, in the segmentation of Water Bodies, there is approximately a 5.46% difference between the weighted OA of the proposed approach and the UNet-ResNet152 network. The improvement of the proposed method compared to others is lowest in the “Others” category. This is likely due to limited data and imbalanced training samples. It can be believed that this imbalance has affected performance, but even in this case, the proposed method still shows better accuracy than the other approaches.

Table 13 presents the weighted evaluation metrics for all architectures. Based on the reported results, it is evident that the proposed approach, including the post-processing step, consistently outperforms the other methods across all metrics. The improvement is particularly notable regarding the MCC, which, given the class imbalance, highlights the method’s effectiveness in enhancing performance under challenging conditions. As a final summary, it appears that adding a post-processing step to a deep learning network, without a significant increase in memory requirements or computational load, helps correct errors in the output of the synergistic semantic classifier and improves the network’s performance across all evaluation parameters.

### 5.5. Statistical Significance Test

To validate the experimental results, a statistical significance test using the Wilcoxon signed-rank scheme was conducted on recall and precision across the different classes. The results, shown in Table 14 top and bottom, indicate that all differences between the proposed method and existing methods, including baseline, are statistically significant. This confirms that the performance improvement achieved by the proposed method is meaningful.

## 6. Conclusions

This study introduced a synergistic semantic segmentation system integrating the DeepLab v3+ deep neural network with a post-processing step. The chosen classes included Pastures, Urban Areas, Other Built-Up Areas, Water Bodies, Grasslands, Forests, Farmland, and Others. Transfer learning was used with DeepLab v3+ pre-trained segmentation networks to optimize the training process and reduce computational demands. In the post-processing step, a dictionary-based K-medoid clustering approach was used. In the K-medoid clustering, spectral codewords were first derived for each class using training data. These spectral codewords, which cover seasonal variations in classes such as Forests and Pastures, were then used to refine the DeepLab v3+ output. The proposed post-processing approach, which involves dictionary training and subsequent use of spectral codewords for performance enhancement, achieved a 1.5% improvement in weighted OA and a 5.7% increase in the weighted MCC compared to the DeepLab v3+ network. It also outperformed selected state-of-the-art semantic segmentation networks, with over a 1.5% in weighted OA and more than a 6% improvement in the weighted MCC.

The main advantages of the proposed method are the ability to analyze vegetation classes based on the data acquisition timing and the addition of a post-processing approach to reduce errors. The two main limitations of the proposed method are computational complexity and inefficiency in cases where deep learning makes errors within a specific region. The second limitation is because of the assumption that errors appear as isolated points within a correct texture. If this assumption is incorrect, the post-processing approach will not check them.

The proposed LCC method has many applications in environmental monitoring, urban expansion tracking, agricultural land management, urban expansion tracking, and water resource management. For example, by segmenting forest areas, the model can track changes in natural landscapes over time and assess patterns of degradation and land use changes caused by human activity. Future work could focus on generalizing the derived codewords for different vegetation types across various regions and creating a spectral dictionary of vegetation classes that can be standardly used in multispectral satellites. Moreover, the post-processing approach could be refined using statistical analysis to define more precise error probability patterns. Another interesting topic is the use of domain adaptation techniques, e.g., feature normalization and fine-tuning, to improve the transferability of the model to different sensors.

## Figures and Tables

**Table 1:** Different bands of the Sentinel-2 satellite images.

Band	Characteristic	Wavelength (μm)	Spatial Resolution (m)
1	Coastal Aerosol (Special Blue)	0.443	60
2	Blue	0.490	10
3	Green	0.560	10
4	Red	0.665	10
5	Vegetation red edge	0.705	20
6	Vegetation red edge	0.740	20
7	Vegetation red edge	0.783	20
8	Near Infrared (NIR)	0.832	10
8A	Narrow NIR	0.865	20
9	Shortwave Infrared (water vapor)	0.945	60
11	Shortwave Infrared	1.610	20
12	Shortwave Infrared	2.190	20

**Table 2:** Key features of CORINE Land Cover 2018 [60].

Feature	Value
Spatial Resolution	100 m
Thematic Accuracy	≥85%
Data Format	Vector and Raster
Geographical Coverage	Entire Europe
Minimum Mapping Unit	25 hectares
Publication Year	2018
Sensors Used	Sentinel-1, Sentinel-2

**Table 3:** Specifications of the used Sentinel-2 images.

Time Range	Cloud Coverage (%)	Image Dimensions at 10 m Resolution	Processed by
20 March to 20 June	3.2	10,980 × 10,980	ESA
21 June to 22 September	4.1	10,980 × 10,980	ESA
23 September to 21 December	2.8	10,980 × 10,980	ESA
22 December to 19 March	1.8	10,980 × 10,980	ESA

**Table 4:** Number of samples per class in the CORINE LC image of the studied area.

Class	Train	Test	Validation
Pastures	41,041,935	12,638,752	13,303,230
Other Built-Up	39,586,397	13,525,201	14,080,078
Water Bodies	58,919,862	19,351,122	19,162,358
Urban Area	81,682,470	27,708,495	27,931,651
Grasslands	235,554,650	77,013,924	80,001,841
Forest	457,877,719	152,133,049	152,919,495
Farmland	555,330,068	187,828,316	182,453,973
Others	18,377,587	5,941,429	6,287,662

**Table 5:** Selected parameters for training the DeepLab v3+ network.

Parameter	Value
trainingOptions	adam
InitialLearnRate	0.005
L2Regularization	0.001
MaxEpochs	200
MiniBatchSize	28
LearnRateSchedule	piecewise
LearnRateDropPeriod	5
LearnRateDropFactor	0.1
Shuffle	every-epoch
VerboseFrequency	20
ExecutionEnvironment	GPU
ValidationData	dsVal
ValidationFrequency	100
OutputNetwork	best-validation-loss
ValidationPatience	100

**Table 6:** Confusion matrix of the DeepLab v3+ model’s output for the test data. (The labels are as follows: (a) Others, (b) Pastures, (c) Other Built-Up, (d) Water Bodies, (e) Urban Area, (f) Grasslands, (g) Forest, and (h) Farmland.)

	(a)	(b)	(c)	(d)	(e)	(f)	(g)	(h)
**(a)**	3,881,463	69,063	102,155	405,707	477,672	20,014	435,430	549,925
**(b)**	685,615	8,805,628	368,464	232,538	844,553	776,759	299,535	625,660
**(c)**	229,845	59,983	9,322,370	1,226,124	694,681	848,189	717,356	426,653
**(d)**	1,268,925	370,458	1,211,953	14,235,666	805,874	788,102	318,736	351,408
**(e)**	409,363	851,682	1,437,711	1,087,237	20,249,132	1,033,593	1,459,924	1,179,853
**(f)**	3,623,855	227,987	1,027,186	3,771,706	2,951,855	60,991,412	1,817,100	2,602,823
**(g)**	2,403,239	1,348,259	2,420,869	187,554	2,985,933	2,107,525	134,510,544	6,169,126
**(h)**	4,235,630	2,042,300	2,312,335	4,135,548	4,960,832	2,622,284	4,631,680	16,288,707

**Table 7:** Distance metrics and their mathematical representations. (The labels are as follows: (a) sqEuclidean, (b) seuclidean, (c) cityblock, (d) chebychev, (e) cosine, and (f) correlation.)

Label	Description	Formula
(a)	Squared Euclidean distance	$\sum_{i=1}^{N}{\left(x_{i}-y_{i}\right)}^{2}$
(b)	Standardized Euclidean distance	$\sum_{i=1}^{N}{\left(Z_{x_{i}}-Z_{y_{i}}\right)}^{2}$
(c)	City block distance	$\sum_{i=1}^{N}\left|x_{i}-y_{i}\right|$
(d)	Chebyshev distance	$\max_{i\in [1\cdots N]}\left(\right|x_{i}-y_{i}\left|\right)$
(e)	The cosine of two non-zero vectors	$\frac{\sum_{i=1}^{N}x_{i}y_{i}}{\sqrt{\sum_{i=1}^{N}x_{i}^{2}}\sqrt{\sum_{i=1}^{N}y_{i}^{2}}}$
(f)	The correlation between two non-zero vectors	$\frac{\sum_{i=1}^{N}\left(x_{i}-\mu_{x}\right)\left(y_{i}-\mu_{y}\right)}{\sqrt{\sum_{i=1}^{N}{\left(x_{i}-\mu_{x}\right)}^{2}}\sqrt{\sum_{i=1}^{N}{\left(y_{i}-\mu_{y}\right)}^{2}}}$

**Table 8:** The number of final medoids and corresponding OA values.

N of Final Medoids	OA
5	73.24
10	77.61
20	82.12
30	85.47
40	87.26
50	88.396
60	88.412
70	88.657
80	88.432
90	88.247
100	88.172

**Table 9:** Confusion matrix for the proposed method on test data. (The labels are as follows: (a) Others, (b) Pastures, (c) Other Built-Up, (d) Water Bodies, (e) Urban Area, (f) Grasslands, (g) Forest, and (h) Farmland.)

	(a)	(b)	(c)	(d)	(e)	(f)	(g)	(h)
**(a)**	3,980,491	62,556	93,585	363,115	453,917	12,564	430,819	544,382
**(b)**	656,595	9,264,039	360,689	112,913	555,553	770,784	296,673	621,506
**(c)**	217,841	49,449	9,807,729	890,426	658,779	815,687	702,871	382,419
**(d)**	727,096	195,466	935,460	16,394,655	442,152	315,996	200,856	139,441
**(e)**	297,316	835,442	1,303,606	480,613	22,731,568	678,017	684,922	697,011
**(f)**	2,744,056	227,018	726,711	1,331,286	1,562,709	66,132,073	1,741,200	2,548,871
**(g)**	1,370,947	1,297,349	1,410,144	47,142	1,809,734	1,914,920	139,781,857	4,500,956
**(h)**	3,489,533	1,876,718	1,774,881	1,221,968	2,680,460	2,499,543	3,806,756	170,478,457

**Table 10:** Recall (%) for different classes using DeepLab v3+ (Top) and the proposed method (bottom). (The labels are as follows: (a) Others, (b) Pastures, (c) Other Built-Up, (d) Water Bodies, (e) Urban Area, (f) Grasslands, (g) Forest, and (h) Farmland.)

**DeepLab v3+ classification errors**								
	**(a)**	**(b)**	**(c)**	**(d)**	**(e)**	**(f)**	**(g)**	**(h)**
**(a)**	65.33	1.16	1.72	6.83	8.04	0.34	7.33	9.26
**(b)**	5.42	69.67	2.92	1.84	6.68	6.15	2.37	4.95
**(c)**	1.7	0.44	68.93	9.07	5.14	6.27	5.3	3.15
**(d)**	6.56	1.91	6.26	73.57	4.16	4.07	1.65	1.82
**(e)**	1.48	3.07	5.19	3.92	73.08	3.73	5.27	4.26
**(f)**	4.71	0.3	1.33	4.9	3.83	79.2	2.36	3.38
**(g)**	1.58	0.89	1.59	0.12	1.96	1.39	88.42	4.06
**(h)**	2.26	1.09	1.23	2.2	2.64	1.4	2.47	86.72
**Proposed method classification errors**								
	**(a)**	**(b)**	**(c)**	**(d)**	**(e)**	**(f)**	**(g)**	**(h)**
**(a)**	67	1.05	1.58	6.11	7.64	0.21	7.25	9.16
**(b)**	5.2	73.3	2.85	0.89	4.4	6.1	2.35	4.92
**(c)**	1.61	0.37	72.51	6.58	4.87	6.03	5.2	2.83
**(d)**	3.76	1.01	4.83	84.72	2.28	1.63	1.04	0.72
**(e)**	1.07	3.02	4.7	1.73	82.04	2.45	2.47	2.52
**(f)**	3.56	0.29	0.94	1.73	2.03	85.87	2.26	3.31
**(g)**	0.9	0.85	0.93	0.03	1.19	1.26	91.88	2.96
**(h)**	1.86	1	0.94	0.65	1.43	1.33	2.03	90.76

**Table 11:** Architectures used for comparison with the proposed model, including total parameters, trainable parameters, and OA.

Architecture	Backbone	Total Parameters	Trainable	Non-Trainable	OA
UNet	ResNet50	32,562,129	32,514,571	47,558	0.8137
UNet	ResNet152	67,296,209	67,150,347	145,862	0.8311
UNet	DenseNet169	19,520,840	19,360,456	160,384	0.7473
FPN	VGG16	17,580,616	17,578,312	2,304	0.7430
LinkNet	MobileNetv2	4,145,592	4,107,144	38,448	0.5981
Deeplab v3+	ResNet50	44,070,592	44,013,792	56,800	0.8362
Proposed	-	44,071,604	44,014,792	56,812	-

**Table 12:** Confusion matrices for the architectures under study: (a) Others, (b) Pastures, (c) Other Built-Up, (d) Water Bodies, (e) Urban Area, (f) Grasslands, (g) Forest, and (h) Farmland, with test data results from (A) UNet-ResNet50 [53], (B) UNet-ResNet152 [53], (C) UNet-DenseNet169 [53], (D) FPN-VGG16 [53], and (E) LinkNet-MobileNetV2 [53].

**(A)**	**(a)**	**(b)**	**(c)**	**(d)**	**(e)**	**(f)**	**(g)**	**(h)**	**Maximum Value**
**(a)**	3734574	224,531	455,254	80,877	101,688	13,597	663,363	667,545	3,734,574
**(b)**	535,685	8,716,979	752,617	332,573	864,752	639,642	490,280	306,224	8,716,979
**(c)**	948,479	769,069	8,546,917	429,519	990,646	269,673	562,898	1,008,000	8,546,917
**(d)**	455,456	1,689,485	124,903	13,930,286	545,270	1,077,397	370,250	1,158,075	13,930,286
**(e)**	1,287,166	1,669,726	2,042,104	598,744	18,957,457	1,541,514	288,534	1,323,250	18,957,457
**(f)**	3,937,202	2,130,370	1,187,036	2,790,107	3,922,274	56,701,374	2,180,020	4,165,541	56,701,374
**(g)**	905,855	2,422,629	3,229,733	3,428,015	2,537,677	5,652,936	133,207,697	748,507	133,207,697
**(h)**	4,732,228	4,913,642	1,692,317	9,520,022	4,532,582	2,488,455	10,558	159,938,512	159,938,512
**(B)**	**(a)**	**(b)**	**(c)**	**(d)**	**(e)**	**(f)**	**(g)**	**(h)**	**Maximum Value**
**(a)**	3,948,325	265,569	419,898	20,372	436,204	63,148	401,975	385,938	3,948,325
**(b)**	244,372	7,982,080	898,318	720,742	308,922	1,023,707	1,078,942	381,669	7,982,080
**(c)**	1,192,477	590,599	9,047,387	837,679	495,974	41,924	353,012	966,149	9,047,387
**(d)**	113,519	696,388	499,815	14,109,665	1,007,550	714,965	1,096,169	1,113,051	14,109,665
**(e)**	228,732	1,518,171	1,179,588	29,808	21,135,472	1,656,782	953,022	1,006,920	21,135,472
**(f)**	3,238,651	3,642,869	1,630,613	360,516	3,125,521	60,025,392	1,413,922	3,576,440	60,025,392
**(g)**	4,511,402	961,521	705,512	4,792,846	240,105	3,740,658	133,184,043	3,996,962	133,184,043
**(h)**	3,154,163	8,135,025	3,313,221	3,655,289	1,302,125	2,707,372	2,607,953	162,953,168	162,953,168
**(C)**	**(a)**	**(b)**	**(c)**	**(d)**	**(e)**	**(f)**	**(g)**	**(h)**	**Maximum Value**
**(a)**	3,367,970	229,274	288,354	379,489	657,135	133,316	808,711	77,180	3,367,970
**(b)**	37,805	7,928,827	937,004	870,874	266,860	845,822	693,289	1,058,271	7,928,827
**(c)**	1,216,751	566,050	8,370,527	483,218	659,784	345,366	1,253,716	629,789	8,370,527
**(d)**	739,805	1,205,271	690,243	13,291,655	311,968	1,468,998	1,002,265	640,917	13,291,655
**(e)**	506,882	1,208,755	1,003,197	1,715,817	19,575,904	163,062	1,961,232	1,573,646	19,575,904
**(f)**	475,771	1,107,585	3,366,194	3,009,838	899,688	59,565,605	4,275,971	4,165,541	59,565,605
**(g)**	5,342,331	720,819	7,431,683	6,778,834	9,244,271	5,652,936	117,194,750	3,210,761	117,194,750
**(h)**	8,551,919	10,005,103	775,997	2,070,073	8,313,678	7,858,470	8,763,951	141,489,125	141,489,125
**(D)**	**(a)**	**(b)**	**(c)**	**(d)**	**(e)**	**(f)**	**(g)**	**(h)**	**Maximum Value**
**(a)**	3,497,084	254,314	365,328	511,483	400,926	312,944	178,598	420,752	3,497,084
**(b)**	546,905	7,866,973	855,360	812,093	865,897	904,630	785,586	1308	7,866,973
**(c)**	156,806	467,406	8,857,601	40,871	760,258	611,428	970,486	1,660,345	8,857,601
**(d)**	630,500	1,602,860	634,964	12,963,903	257,583	947,003	1,597,594	716,715	12,963,903
**(e)**	1,844,027	1,322,699	1,390,920	2,789,095	17,981,969	245,215	821,801	1,312,769	17,981,969
**(f)**	4,479,153	2,394,133	2,232,438	3,808,280	2,388,059	54,937,563	2,670,602	5,930,120	54,937,563
**(g)**	5,582,821	2,628,237	979,879	771,780	10,175,248	4,393,376	119,456,135	9,868,347	119,456,135
**(h)**	8,027,193	8,626,731	1,284,711	3,622,669	9,018,160	5,930,120	8,244,210	143,074,522	143,074,522
**(E)**	**(a)**	**(b)**	**(c)**	**(d)**	**(e)**	**(f)**	**(g)**	**(h)**	**Maximum Value**
**(a)**	2,832,178	13,213	583,996	216,669	494,508	679,733	569,497	551,635	2,832,178
**(b)**	649,146	6,308,361	1,140,412	27,057	1,618,582	2,546,825	253,749	94,620	6,308,361
**(c)**	700,088	25,275	6,326,817	2,310,329	398,321	2,494,454	270,397	999,520	6,326,817
**(d)**	1,569,608	1,790,894	1,738,175	10,049,766	1,162,407	374,869	2,016,868	648,535	10,049,766
**(e)**	3,061,373	301,256	3,470,979	112,332	14,617,573	2,982,928	2,984,030	178,024	14,617,573
**(f)**	7,084,763	2,414,793	6,057,897	4,517,497	5,931,353	44,632,108	6,003,199	372,314	44,632,108
**(g)**	8,645,276	6,478,172	12,870,898	9,902,570	7,512,575	3,955,315	99,379,893	3,388,350	99,379,893
**(h)**	8,300,278	15,158,702	1,826,825	15,968,780	513,268	16,703,746	16,718,218	112,638,499	112,638,499

**Table 13:** Evaluation metrics (%) for the studied architectures and the proposed method.

		Precision	Recall	OA	F1-Score	MCC
**(a) Others**	UNet-ResNet50 [53]	22.58	62.86	96.97	33.23	36.51
	UNet-ResNet152 [53]	23.74	66.45	97.04	34.98	38.60
	UNet-DenseNet169 [53]	13.73	56.69	95.22	22.11	26.28
	FPN-VGG16 [53]	14.12	58.86	95.22	22.78	27.23
	LinkNet-MobileNetv2 [53]	8.62	47.67	93.32	14.60	18.18
	Deeplab V3+ [13]	23.19	65.33	96.99	34.23	37.78
	Proposed	29.52	67.00	97.69	40.98	43.52
**(b) Pastures**	UNet-ResNet50 [53]	38.68	68.97	96.42	49.56	50.02
	UNet-ResNet152 [53]	33.55	63.16	95.87	43.82	44.16
	UNet-DenseNet169 [53]	34.52	62.73	96.02	44.53	44.71
	FPN-VGG16 [53]	31.26	62.24	95.55	41.62	42.33
	LinkNet-MobileNetv2 [53]	19.42	49.91	93.35	27.96	28.34
	Deeplab V3+ [13]	63.92	69.67	98.23	66.67	65.63
	Proposed	67.09	73.30	98.40	70.06	69.31
**(c) Other Built-Up**	UNet-ResNet50 [53]	47.40	63.19	97.09	54.17	53.28
	UNet-ResNet152 [53]	51.13	66.89	97.35	57.96	57.16
	UNet-DenseNet169 [53]	36.61	61.89	96.04	46.01	45.74
	FPN-VGG16 [53]	53.36	65.49	97.50	58.80	57.85
	LinkNet-MobileNetv2 [53]	18.60	46.78	92.97	26.62	26.45
	Deeplab V3+ [13]	51.21	68.93	97.36	58.76	58.11
	Proposed	59.76	72.51	97.92	65.52	64.78
**(d)** **Water** **Bodies**	UNet-ResNet50 [53]	44.78	71.99	95.44	55.21	54.61
	UNet-ResNet152 [53]	57.53	72.91	96.84	64.31	63.17
	UNet-DenseNet169 [53]	55.31	68.69	96.61	61.28	59.91
	FPN-VGG16 [53]	51.20	66.99	96.22	58.04	56.66
	LinkNet-MobileNetv2 [53]	23.31	51.93	91.46	32.18	30.93
	Deeplab V3+ [13]	56.31	73.57	96.74	63.79	62.72
	Proposed	78.66	84.72	98.51	81.58	80.86
**(e)** **Urban** **Area**	UNet-ResNet50 [53]	58.42	68.42	95.52	63.02	60.87
	UNet-ResNet152 [53]	75.34	76.28	97.28	75.81	74.37
	UNet-DenseNet169 [53]	49.03	70.65	94.26	57.88	55.97
	FPN-VGG16 [53]	42.97	64.90	93.23	51.70	49.41
	LinkNet-MobileNetv2 [53]	45.33	52.75	93.81	48.76	45.63
	Deeplab V3+ [13]	59.61	73.08	95.73	65.66	63.78
	Proposed	73.58	82.04	97.35	77.58	76.30
**(f)** **Grasslands**	UNet-ResNet50 [53]	82.92	73.62	93.55	77.99	74.41
	UNet-ResNet152 [53]	85.78	77.94	94.57	81.67	78.62
	UNet-DenseNet169 [53]	77.20	77.34	92.94	77.27	73.09
	FPN-VGG16 [53]	82.54	71.33	93.21	76.53	72.85
	LinkNet-MobileNetv2 [53]	60.01	57.95	87.48	58.97	51.59
	Deeplab V3+ [13]	88.15	79.20	95.12	83.43	80.74
	Proposed	90.42	85.87	96.39	88.09	86.00
**(g)** **Forest**	UNet-ResNet50 [53]	96.69	87.56	95.27	91.90	88.78
	UNet-ResNet152 [53]	94.40	87.54	94.59	90.84	87.13
	UNet-DenseNet169 [53]	88.98	77.03	90.03	82.58	76.03
	FPN-VGG16 [53]	87.55	78.52	89.99	82.79	75.98
	LinkNet-MobileNetv2 [53]	77.52	65.32	83.56	70.90	59.99
	Deeplab V3+ [13]	93.29	88.42	94.95	90.79	86.93
	Proposed	94.67	91.88	95.93	93.26	90.36
**(h)** **Farmland**	UNet-ResNet50 [53]	94.46	85.15	92.49	89.57	84.00
	UNet-ResNet152 [53]	93.45	86.76	92.68	89.98	84.37
	UNet-DenseNet169 [53]	92.50	75.33	88.35	83.04	75.21
	FPN-VGG16 [53]	89.74	76.17	87.68	82.40	73.60
	LinkNet-MobileNetv2 [53]	94.76	59.97	83.59	73.45	65.85
	Deeplab V3+ [13]	93.19	86.72	92.57	89.84	84.13
	Proposed	94.76	90.76	94.60	92.72	88.48

**Table 14:** Statistical significance of differences in recall (top) and precision (bottom) between the compared models: (a) UNet-ResNet50, (b) UNet-ResNet152, (c) UNet-DenseNet169, (d) FPN-VGG16, (e) LinkNet-MobileNetv2, (f) Deeplab, and (g) DeeplabP (TRUE = significant difference).

Recall Comparisons							
	(a)	(b)	(c)	(d)	(e)	(f)	(g)
**(a)**	TRUE	FALSE	FALSE	TRUE	TRUE	TRUE	TRUE
**(b)**	FALSE	FALSE	TRUE	TRUE	TRUE	FALSE	TRUE
**(c)**	FALSE	TRUE	FALSE	FALSE	TRUE	TRUE	TRUE
**(d)**	TRUE	TRUE	FALSE	FALSE	TRUE	TRUE	TRUE
**(e)**	TRUE	TRUE	TRUE	TRUE	FALSE	TRUE	TRUE
**(f)**	TRUE	FALSE	TRUE	TRUE	TRUE	FALSE	TRUE
**(g)**	TRUE	TRUE	TRUE	TRUE	TRUE	TRUE	FALSE
**Precision Comparisons**							
	**(a)**	**(b)**	**(c)**	**(d)**	**(e)**	**(f)**	**(g)**
**(a)**	TRUE	FALSE	FALSE	FALSE	TRUE	FALSE	TRUE
**(b)**	FALSE	FALSE	TRUE	TRUE	TRUE	FALSE	TRUE
**(c)**	FALSE	TRUE	FALSE	FALSE	TRUE	TRUE	TRUE
**(d)**	FALSE	TRUE	FALSE	FALSE	TRUE	TRUE	TRUE
**(e)**	TRUE	TRUE	TRUE	TRUE	FALSE	TRUE	TRUE
**(f)**	FALSE	FALSE	TRUE	TRUE	TRUE	FALSE	TRUE
**(g)**	TRUE	TRUE	TRUE	TRUE	TRUE	TRUE	FALSE

## Data Availability

This study was developed using publicly available data, fully identified in the manuscript.
